# Photorespiration Is Associated with Nitrogen Metabolism and Nitrogen Productivity in Kentucky Bluegrass

**DOI:** 10.3390/biology15181628

**Published:** 2026-09-16

**Authors:** Xiaoyan Li, Huisen Zhu, Dina Li, Tao Xu, Huifang Cen, Yan Zhang

**Affiliations:** College of Grassland Science, Shanxi Agriculture University, Taigu, Jinzhong 030801, China; lixiaoyan_199098@163.com (X.L.); 13354593936@163.com (D.L.); xutao@sxau.edu.cn (T.X.); cenhuifangqk@163.com (H.C.); xmszhangyan@126.com (Y.Z.)

**Keywords:** Kentucky bluegrass, photorespiration, nitrogen metabolism, PFP_N_, *PpSHMT*

## Abstract

Photorespiration links carbon metabolism with nitrogen assimilation in C_3_ plants, but its relationship with nitrogen productivity in perennial turfgrass remains unclear. Ten Kentucky bluegrass (*Poa pratensis* L.) cultivars were screened for photorespiration rate and intensity. ‘Heidi’ and ‘My Holiday’ exhibited the highest and lowest photorespiration intensities (31.17 ± 2.75% and 6.05 ± 1.15%, respectively), and their absolute photorespiration rates were 2.36 ± 0.22 and 0.49 ± 0.09 μmol CO_2_·m^−2^·s^−1^, representing a 4.8-fold difference. Under baseline conditions, ‘Heidi’ showed higher NDVI, shoot N concentration, and N accumulation, as well as component-specific differences in selected epidermal traits and biochemical measurements, whereas the measured gas-exchange variables were unchanged. Nitrogen treatments further showed that cultivar responses depended on nitrogen form and application rate. Under low nitrate supply, ‘My Holiday’ had a higher photorespiration rate than ‘Heidi’, whereas their partial factor productivity of applied nitrogen based on shoot dry matter (PFP_N_) did not differ significantly. Comparative transcriptomic analysis identified serine hydroxymethyltransferase 4 (*SHM4*, hereafter *PpSHMT*) as a photorespiration-related candidate transcript, with a log_2_ fold change of 3.37 in ‘Heidi’ relative to ‘My Holiday’. qRT-PCR confirmed higher *PpSHMT* transcript abundance in ‘Heidi’, but bulk-leaf SHMT relative protein abundance did not differ significantly between the cultivars. Transient delivery of pBI121-PpSHMT into *Nicotiana tabacum* L. cv. K326 leaves was associated with changes in selected biochemical measurements. Overall, the results demonstrate a treatment-dependent association between photorespiration and nitrogen productivity and identify *PpSHMT* as a candidate requiring further functional investigation.

## Simple Summary

Photorespiration links carbon metabolism with nitrogen assimilation in C_3_ plants, but its relationship with nitrogen productivity in perennial turfgrass remains unclear. In this study, we selected ‘Heidi’ and ‘My Holiday’, which exhibited the highest and lowest photorespiration intensities from ten Kentucky bluegrass (*Poa pratensis* L.) cultivars. Under baseline conditions, ‘Heidi’ showed higher NDVI, shoot N concentration, and N accumulation, as well as component-specific differences in selected epidermal traits and biochemical measurements, whereas the measured gas-exchange variables were unchanged. Nitrogen treatments further showed that cultivar responses depended on nitrogen form and application rate. Under low nitrate supply, ‘My Holiday’ had a higher photorespiration rate than ‘Heidi’, whereas their partial factor productivity of applied nitrogen based on shoot dry matter (PFP_N_) did not differ significantly. Comparative transcriptomic analysis identified serine hydroxymethyltransferase 4 (SHM4, hereafter *PpSHMT*) as a photorespiration-related candidate transcript. qRT-PCR confirmed higher *PpSHMT* transcript abundance in ‘Heidi’, but bulk-leaf SHMT relative protein abundance did not differ significantly between the cultivars. Transient delivery of pBI121-PpSHMT into *Nicotiana tabacum* L. cv. K326 leaves was associated with changes in selected biochemical measurements. Overall, the results demonstrate a treatment-dependent association between photorespiration and nitrogen productivity and identify *PpSHMT* as a candidate requiring further functional investigation.

## 1. Introduction

Kentucky bluegrass (*P. pratensis* L.) is among the most widely cultivated cool-season turfgrasses in temperate regions, valued for its rhizomatous growth, dense sod, and rapid recovery from wear [1]. However, maintaining its aesthetic and functional quality typically requires substantial nitrogen (N) fertilizer, and Kentucky bluegrass ranks among the more N-demanding turfgrass species commonly grown for lawns, athletic fields, and golf-course roughs. Only a fraction of applied N is recovered as harvestable or aboveground biomass, so intensive fertilization of amenity turf disproportionately contributes to nitrate leaching, nitrous oxide emissions, and rising input costs [2,3]. Identifying cultivars and physiological traits associated with greater nitrogen productivity has therefore become an increasingly important breeding objective for cool-season turfgrasses [4].

Carbon assimilation in C_3_ species such as Kentucky bluegrass is inherently constrained by the dual carboxylase/oxygenase activity of Rubisco [5,6]. When Rubisco fixes O_2_ rather than CO_2_, the resulting 2-phosphoglycolate is detoxified via the photorespiratory pathway, a cyclical process spanning the chloroplast, peroxisome, and mitochondrion [7]. Photorespiration has long been viewed as an energetically costly process that can substantially reduce net photosynthetic efficiency, depending on temperature, irradiance, and water availability [8,9]. Yet a growing body of evidence indicates that photorespiration is not merely a carbon-wasting side reaction but a central metabolic hub that buffers cellular redox balance, supplies precursors for one-carbon and serine metabolism, and interfaces directly with primary nitrogen and sulfur assimilation [10,11,12].

The mechanistic link between photorespiration and nitrogen metabolism centers on the release and reassimilation of ammonia. During the glycine decarboxylase (GDC)-catalyzed conversion of glycine to serine, each turn of the photorespiratory cycle releases NH_3_ at a flux that can substantially exceed the rate of primary nitrate or ammonium assimilation [12]. This photorespiratory ammonia is recaptured by the glutamine synthetase/glutamate synthase (GS/GOGAT) cycle, the same enzymatic system responsible for primary nitrogen assimilation, thereby coupling photorespiratory flux directly to the whole-plant nitrogen economy [2,13]. Enzymes such as glutamate: glyoxylate aminotransferase (GGAT) and serine: glyoxylate aminotransferase (SGAT) further connect carbon skeletons recycled through photorespiration to amino acid synthesis, while serine hydroxymethyltransferase (SHMT) catalyzes the reversible interconversion of glycine and serine and synchronizes photorespiratory flux with one-carbon metabolism [14,15]. Recent metabolic engineering work has shown that manipulating enzymes in the serine biosynthesis and photorespiratory networks can measurably alter whole-plant nitrogen content and assimilation capacity, providing evidence that photorespiration can influence plant nitrogen status [16].

Despite this expanding mechanistic understanding, most evidence linking photorespiration to nitrogen metabolism has been obtained from model species or major grain crops, and the relationship remains less well characterized in perennial forage and turfgrass species managed primarily for vegetative rather than reproductive biomass. Previous research in Kentucky bluegrass has characterized canopy photosynthesis, ecosystem respiration, and carbon balance under contrasting light conditions [17]. Genotypic variation in nitrogen productivity and tolerance of low-N conditions has also been documented [4,18], and canopy indices such as the Normalized Difference Vegetation Index (NDVI) have been widely used as convenient, non-destructive proxies for leaf nitrogen status [19,20]. However, these studies did not directly compare photorespiration intensity among Kentucky bluegrass cultivars or examine how cultivar-level variation in photorespiration is related to nitrogen-metabolism-related traits and nitrogen productivity. This relationship therefore remains insufficiently understood in perennial turfgrass.

In rice, the mitochondrial serine hydroxymethyltransferase encoded by *OsSHM1* is required for normal photorespiratory metabolism: an *osshm1* mutant showed a lethal chlorotic phenotype under ambient CO_2_ that was rescued by elevated CO_2_ [21]. More recently, engineering a chloroplast-targeted photorespiratory bypass in rice improved photosynthetic efficiency, biomass, yield, and nitrogen uptake [22]. Together, these studies provide a rice-specific basis for examining photorespiratory variation and its association with nitrogen productivity in C_3_ grasses. However, direct evidence linking cultivar-level variation in a specific SHMT transcript with nitrogen productivity remains limited, particularly in perennial cool-season turfgrasses.

To address these gaps, we first quantified photorespiration rate and photorespiration intensity in ten Kentucky bluegrass cultivars under light-saturating conditions. ‘Heidi’ and ‘My Holiday’, which exhibited the highest and lowest photorespiration intensities, respectively, were then selected for comparative physiological, anatomical, biochemical, and nitrogen-related analyses. Comparative transcriptomics was subsequently used to identify candidate transcripts associated with their contrasting photorespiratory phenotypes. *PpSHMT* was not selected a priori but emerged from this comparative analysis as a photorespiration-related candidate transcript. It was subsequently assessed by *Agrobacterium*-mediated transient delivery in tobacco. This integrated approach was designed to examine whether the association between photorespiration and nitrogen productivity was consistent across nitrogen forms and application rates and to evaluate *PpSHMT* as a candidate potentially linking photorespiration with nitrogen metabolism in Kentucky bluegrass.

## 2. Materials and Methods

### 2.1. Bluegrass Cultivars and Their Growth and Maintenance

Kentucky bluegrass cultivars were grown in a garden soil substrate (Table 1) with the following properties: pH 7.35, total salt content 0.0424%, available phosphate 19.27 mg·kg^−1^, total phosphate 0.642 g·kg^−1^, alkali-hydrolyzable nitrogen 143.08 mg·kg^−1^, total nitrogen 0.994 g·kg^−1^, available potassium 157.14 mg·kg^−1^, and organic matter 20.67 g·kg^−1^. Plants were maintained in a greenhouse under controlled conditions (mean temperature 25 °C; relative humidity 60%). Experimental pots were plastic flowerpots with an inner diameter of 29 cm at the top, a bottom diameter of 18.5 cm, and a height of 23 cm. All measurements were conducted after three months of growth. Leaf samples were collected with scissors at a height of 5 cm above the soil surface. Ten Kentucky bluegrass cultivars were randomly arranged in the greenhouse, with three biological replicates per cultivar.

To examine whether cultivar responses depended on nitrogen form and application rate, ‘Heidi’ and ‘My Holiday’ plants were transplanted into pots measuring 7.5 cm in diameter and 25 cm in height, with 15 plants per pot. Each pot constituted one independent experimental unit. The substrate consisted of washed sand and nutrient soil at a ratio of 9:1. Plants were acclimated for 7 days in a controlled-environment chamber under a 16 h light/8 h dark photoperiod, a light intensity of 20,000 lx, a temperature of 25 ± 1 °C, a CO_2_ concentration of 400 μmol·mol^−1^, and a relative humidity of 60–70%.

Seven nitrogen treatments were applied to each cultivar: a zero-N control (N0); ammonium supplied as (NH_4_)_2_SO_4_ at 1, 5, or 10 g elemental N·m^−2^ (LAN, MAN, and HAN, respectively); and nitrate supplied as Ca(NO_3_)_2_·4H_2_O at the same three elemental-N rates (LNN, MNN, and HNN, respectively). The surface area of each pot was 0.004418 m^2^. The three rates therefore corresponded to 0.004418, 0.02209, and 0.04418 g elemental N per pot. The corresponding fertilizer-compound masses were 0.02084, 0.1041, and 0.2084 g (NH_4_)_2_SO_4_ per pot and 0.03724, 0.1862, and 0.3724 g Ca(NO_3_)_2_·4H_2_O per pot.

The experiment comprised one zero-N treatment and six nitrogen-form × nitrogen-rate combinations for each cultivar. Four pots were initially established for each cultivar–treatment combination, whereas measurements and subsequent analyses were based on three pots selected for comparable growth (*n* = 3). Plants were sampled 21 days after initiation of the nitrogen treatments. Each pot received 40 mL of deionized water every 3 days.

### 2.2. Sampling and Parameter Measurement

A portable photosynthesis system (LI-6400XT; LI-COR Inc., Lincoln, NE, USA) was used to measure photosynthetic parameters in Kentucky bluegrass leaves. Each leaf was assessed under low-oxygen (2%) and ambient-oxygen (21%) conditions [22,23,24]. Photorespiration rate (Rp) was calculated as the difference between the net photosynthetic rates measured under 2% and 21% O_2_. Photorespiration intensity was calculated as Rp/Pn × 100%, where Pn represents the net photosynthetic rate measured under ambient O_2_ (21%). Photorespiration intensity was used as the primary criterion for selecting cultivars with contrasting photorespiratory phenotypes. Measurements were conducted at a light intensity of 1200 μmol·m^−2^·s^−1^, a CO_2_ concentration of 400 μmol·mol^−1^ in the leaf chamber, a temperature of 25 °C, and an airflow rate of 500 μmol·s^−1^. Net photosynthetic rate (Pn), intercellular CO_2_ concentration (Ci), stomatal conductance (Gs), and transpiration rate (Tr) were recorded. For each measurement, two leaves were placed side by side and inserted into the leaf chamber. Each pot was measured twice, and the measurement was repeated three times.

The Normalized Difference Vegetation Index (NDVI) was determined using a Turf Colour Meter (TCM 500 NDVI system; Spectrum Technologies Inc., Aurora, IL, USA), and the measurement was repeated three times. The handheld device was positioned directly on or near the turf surface to measure reflectance at 660 nm (red) and 850 nm (near-infrared). NDVI was calculated as:

(1) NDVI=(R850−R660)/(R850+R660),

where *R* represents reflectance at the respective wavelengths.

Leaf samples were rinsed with distilled water and used to prepare freehand sections for temporary slides. Epidermal structures were observed and photographed using an Olympus BX51 optical microscope, and measurements were performed using cellSens Dimension (1.18). For permanent sections, 5 mm × 5 mm leaf segments from the middle region were fixed in FAA solution (formalin:glacial acetic acid:70% ethanol = 5:5:90). Paraffin-embedded sections were prepared following an improved protocol and examined under the Olympus BX51; Olympus Corporation, Tokyo, Japan [25,26]. Fifty microscopic fields were examined for each cultivar and treated as observational subsamples. The three biological replicates (*n* = 3), rather than the individual microscopic fields, were used as the independent units for statistical analysis.

Chlorophyll a, chlorophyll b, and carotenoids were extracted with acetone and quantified as described by Kaur et al. [27]. Total nitrogen content in shoots and soil was determined using the Kjeldahl digestion method [28]. Shoot nitrogen accumulation was calculated from shoot dry mass per pot and shoot N concentration and was expressed as mg N·pot^−1^. For the nitrogen-treatment experiment, the partial factor productivity of applied nitrogen based on shoot dry matter (PFP_N_) was calculated by dividing the shoot dry mass collected 21 days after treatment by the total amount of elemental N applied to the corresponding pot and was expressed as kg shoot dry matter·kg^−1^ N [29]. Applied N was calculated on an elemental-N basis rather than from the mass of (NH_4_)_2_SO_4_ or Ca(NO_3_)_2_·4H_2_O, and PFP_N_ was not calculated for N0 because no N was applied.

The relative protein abundance of Rubisco, SGAT, GGAT, SHMT, GDC, and HPR was measured using commercial plant ELISA kits (Shanghai Enzyme-linked Biotechnology Co., Ltd., Shanghai, China): Rubisco (YJ402362), SGAT (YJ406605), GGAT (YJ406250), SHMT (YJ405542), GDC (YJ406591), and HPR (YJ405996). Fresh leaf tissue (0.1 g) was homogenized on ice, and the supernatant was collected. Absorbance was measured at 450 nm. Standard curves were fitted within the linear response range (R^2^ > 0.99), and corrected sample absorbance values within this range were used to calculate the supernatant concentrations, which were converted to relative protein abundance on a fresh-weight basis (U·g^−1^ FW).

GO, GS, GOGAT, and GDH activities were measured using microplate colorimetric assay kits from the same manufacturer. For GO, the total absorbance increase over 3 min at 324 nm and 25 °C (ΔA_3_) was recorded, and activity was calculated as 784 × ΔA_3_/W and expressed as nmol·min^−1^·g^−1^ FW, where W is the sample fresh weight. GS activity was determined from the absorbance difference between the measurement and control wells at 540 nm and calculated as 190 × ΔA/W. One unit was defined as the amount of enzyme generating 1 μmol γ-glutamyl hydroxamate per minute, and GS activity was expressed as U·g^−1^ FW, equivalent to μmol·min^−1^·g^−1^ FW. GOGAT and GDH activities were determined from the total absorbance change over 5 min at 340 nm and 25 °C and expressed as nmol·min^−1^·g^−1^ FW after correction for the reaction system. Three biological replicates were analyzed for each measurement.

### 2.3. Photorespiration-Related Transcriptome Analysis and Transient Delivery of PpSHMT in Tobacco

#### 2.3.1. Plant Materials and Their Origin

Young and fully expanded leaves were collected independently from three pots of each cultivar, with each pot constituting one biological replicate. The samples were processed separately throughout RNA extraction, library construction, and sequencing, resulting in three independent libraries per cultivar and six libraries in total. Leaf tissue from the middle region was excised, immediately frozen in liquid nitrogen, and stored at −80 °C until analysis. *N. tabacum* L. cv. K326 was obtained from the College of Grassland Science Germplasm Resource Bank at Shanxi Agricultural University.

#### 2.3.2. Experimental Treatments and Measurement Methods

Total RNA was extracted from three biological replicates of each cultivar using the TRIzol method. Poly(A)-containing mRNA was enriched using oligo(dT) magnetic beads, fragmented, and reverse-transcribed to construct cDNA libraries. The libraries were sequenced by Hangzhou Lianchuan Biotechnology Co., Ltd. on the Illumina NovaSeq 6000; Illumina, Inc., San Diego, CA, USA platform using 150 bp paired-end reads.

Adapter sequences and low-quality regions were removed using Cutadapt (1.9)and fqtrim (0.94). Reads shorter than 100 bp after trimming or containing more than 5% of undetermined bases were discarded. Clean reads from the six libraries were combined only to generate the de novo reference transcriptome using Trinity, whereas the libraries were retained separately for expression quantification and differential-expression analysis. Transcript abundance in each library was estimated using Salmon and expressed as transcripts per million (TPM). Differential-expression analysis was performed using edgeR (3.40.2) based on read-count data. Transcripts with |log_2_FC| ≥ 1 and a false discovery rate (FDR) < 0.05 were considered differentially expressed, with Benjamini–Hochberg correction applied for multiple testing.

For qRT-PCR validation, first-strand cDNA was synthesized using the Evo M-MLV Reverse Transcription Kit II (Accurate Biotechnology (Hunan) Co., Ltd., Changsha, China). The primers were PpSHMT-F (5′-GAGAACTTCACCTCCCTCGCCG-3′), PpSHMT-R (5′-CGGCAGAGGTTTTCGACCTGGT-3′), *Actin*-F (5′-TGTTGGATTCTGGTGATGGTGTC-3′), and *Actin*-R (5′-AGGATGGCGTGCGGAAGG-3′). Each 20-μL reaction contained 10 μL of 2× Green qPCR MasterMix, 0.5 μL of each primer, 2 μL of cDNA template, and 7 μL of RNase-free water. Amplification consisted of 95 °C for 2 min, followed by 40 cycles of 95 °C for 15 s and 60 °C for 30 s, followed by melting-curve analysis. Actin was used as the reference gene, and relative *PpSHMT* transcript abundance was calculated using the 2^−ΔΔCt^ method. Three biological replicates were analyzed for each cultivar, with three technical replicates per biological sample.

The *PpSHMT* coding sequence was amplified and cloned into the pTOPO Blunt vector (Beijing Aidelai Biotechnology Co., Ltd., Beijing, China). After verification by Sanger sequencing, the insert was introduced into pBI121 using the BamHI and KpnI restriction sites to generate pBI121-PpSHMT. The recombinant construct was transferred into *Agrobacterium tumefaciens* GV3101 by the freeze–thaw method and infiltrated into leaves of *N. tabacum* L. cv. K326 following established procedures [30,31].

Tobacco plants were grown in a 1:1 mixture of vermiculite and nutrient soil at 25 °C and 60% relative humidity under a 16 h light/8 h dark photoperiod. Untreated wild-type leaves served as the control group (CK), whereas leaves infiltrated with *A. tumefaciens* GV3101 carrying pBI121-PpSHMT constituted the transient-delivery group (TG). No pBI121 empty-vector infiltration control was included. Three biological replicates were analyzed in each group.

### 2.4. Data Analysis

Statistical analyses were performed using SPSS 27 (IBM, Armonk, NY, USA). Photorespiration rate and photorespiration intensity among the ten cultivars were analyzed separately using one-way analysis of variance followed by Tukey’s HSD test. Two-sided independent-samples *t*-tests were used for baseline comparisons of physiological, anatomical, and biochemical traits between ‘Heidi’ and ‘My Holiday’.

For the qRT-PCR analysis, the mean Ct value of the three technical replicates was calculated for each biological replicate. Cultivar differences were tested using the ΔCt values from three biological replicates per cultivar and a two-sided independent-samples *t*-test. Relative expression values calculated using the 2^−ΔΔCt^ method were used for graphical presentation.

The nitrogen-treatment experiment was analyzed separately. Photorespiration rate across all seven nitrogen treatments was evaluated using a two-way analysis of variance with cultivar and nitrogen treatment as fixed factors. For the six fertilized treatments, photorespiration rate and PFP_N_ were additionally analyzed using a three-way analysis of variance with cultivar, nitrogen form, and nitrogen rate as fixed factors. Following a significant interaction, cultivar differences within individual nitrogen treatments were evaluated using two-sided independent-samples *t*-tests. Holm adjustment was applied separately to the seven photorespiration-rate comparisons and the six PFP_N_ comparisons.

Each pot containing 15 plants constituted one independent experimental unit, and three pots were analyzed for each cultivar–treatment combination. Unless otherwise specified, data are presented as the mean ± SD. Statistical significance was determined at *p* < 0.05, with Holm-adjusted *p* < 0.05 used for the treatment-specific cultivar comparisons.

## 3. Results

### 3.1. Photorespiration Rate and Intensity of Ten Kentucky Bluegrass Cultivars

Photorespiration rate and photorespiration intensity were quantified under light-saturating conditions across ten Kentucky bluegrass cultivars, revealing substantial genotypic variation (Table 2). ‘Jackpot’ exhibited the highest absolute photorespiration rate (3.48 ± 0.20 μmol CO_2_·m^−2^·s^−1^), whereas ‘My Holiday’ exhibited the lowest (0.49 ± 0.09 μmol CO_2_·m^−2^·s^−1^). By contrast, ‘Heidi’ showed the highest photorespiration intensity (31.17 ± 2.75%), while ‘My Holiday’ showed the lowest (6.05 ± 1.15%). Because photorespiration intensity normalizes photorespiratory CO_2_ release to net photosynthesis, it was used as the primary criterion for identifying contrasting photorespiratory phenotypes. Accordingly, ‘Heidi’ and ‘My Holiday’ were selected as the high- and low-photorespiration-intensity cultivars, respectively. Their absolute photorespiration rates were 2.36 ± 0.22 and 0.49 ± 0.09 μmol CO_2_·m^−2^·s^−1^, respectively, representing a 4.8-fold difference.

### 3.2. Photosynthetic Indices and Leaf Microstructure of Two Kentucky Bluegrass Cultivars

**Figure 1:**
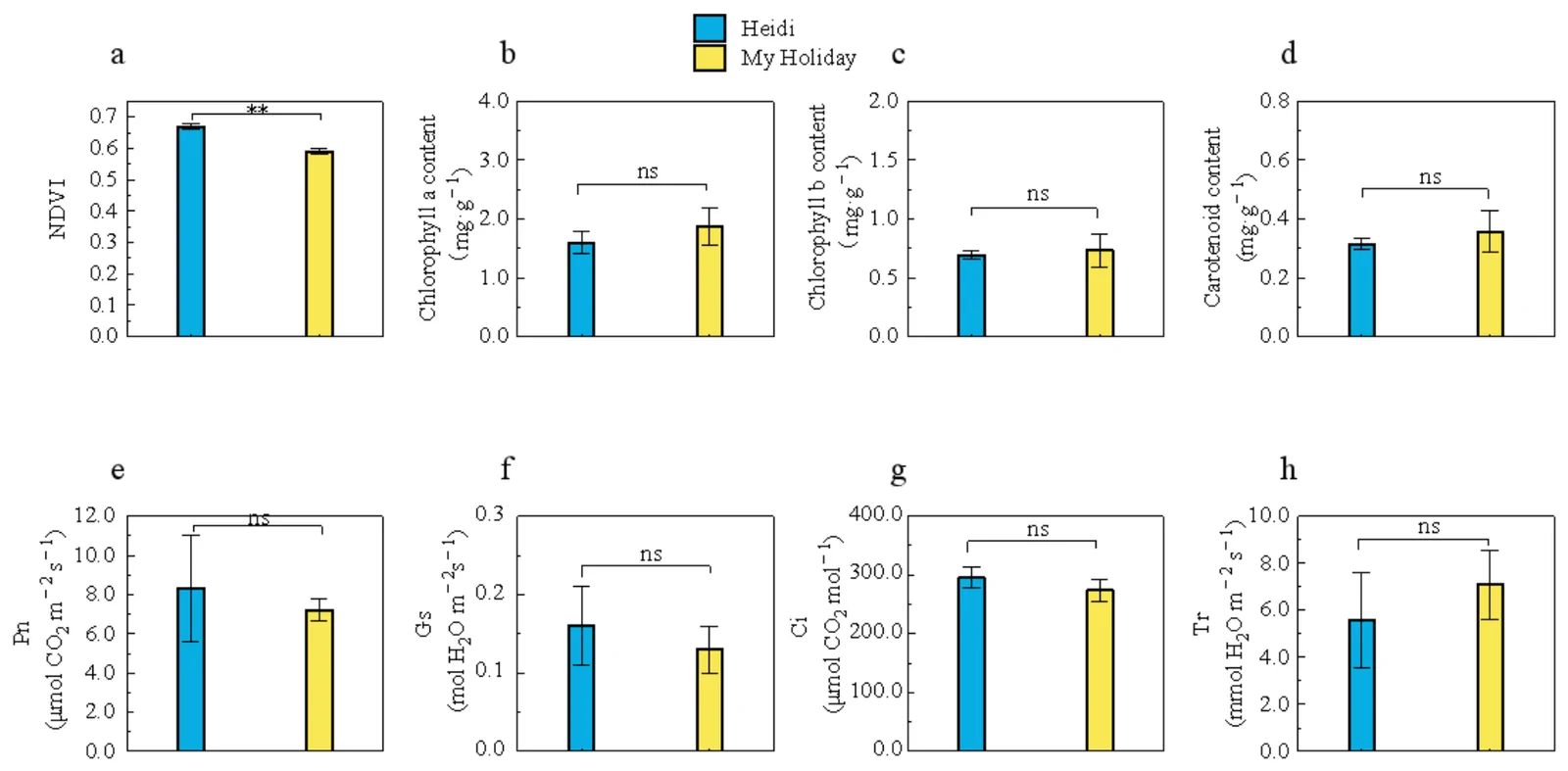
NDVI, pigment contents, and photosynthetic gas-exchange parameters of two Kentucky bluegrass cultivars. (**a**) NDVI; (**b**) chlorophyll a content; (**c**) chlorophyll b content; (**d**) carotenoid content; (**e**) net photosynthetic rate (Pn); (**f**) stomatal conductance (Gs); (**g**) intercellular CO_2_ concentration (Ci); and (**h**) transpiration rate (Tr). **, indicates significant differences between cultivars at *p* < 0.01; ns indicates no significant difference (*p* ≥ 0.05; two-sided independent-samples *t*-test).

To determine whether contrasting photorespiration intensities were associated with differences in photosynthetic performance and leaf structure, NDVI, pigment contents, photosynthetic parameters, and microanatomical traits were compared between ‘Heidi’ (high photorespiration intensity) and ‘My Holiday’ (low photorespiration intensity). NDVI differed significantly between the two cultivars (*p* < 0.01), with ‘Heidi’ showing a markedly higher NDVI value, indicating a denser or more vigorous canopy (Figure 1a). In contrast, chlorophyll a, chlorophyll b, and carotenoid contents did not differ significantly between cultivars (Figure 1b–d). Similarly, no significant differences were observed in net photosynthetic rate (Pn), stomatal conductance (Gs), intercellular CO_2_ concentration (Ci), or transpiration rate (Tr) (Figure 1e–h).

Several leaf epidermal traits differed significantly between ‘Heidi’ and ‘My Holiday’ (Table 3). In the upper epidermis, ‘Heidi’ had a higher cell density than ‘My Holiday’ (*p* = 0.004), whereas ‘My Holiday’ had a higher stomatal length-to-width ratio (*p* = 0.043) and stomatal index (*p* = 0.003). Upper-epidermal stomatal density did not differ significantly between the cultivars (*p* = 0.283). In the lower epidermis, stomatal density was significantly higher in ‘Heidi’ than in ‘My Holiday’ (*p* = 0.003), whereas the other measured lower-epidermal traits did not differ significantly between the cultivars (*p* > 0.05).

‘Heidi’ had significantly thicker upper and lower epidermal layers than ‘My Holiday’ (*p* = 0.024 and *p* = 0.015, respectively; Table 3). Although total leaf thickness, sclerenchyma thickness, and vascular bundle area were numerically higher in ‘Heidi’, none of these differences were statistically significant (*p* = 0.157, *p* = 0.151, and *p* = 0.138, respectively). These results identify specific cultivar-level differences in epidermal structure without indicating uniform differences across all measured anatomical traits.

### 3.3. Nitrogen Metabolism and Photorespiration in Kentucky Bluegrass

**Figure 2:**
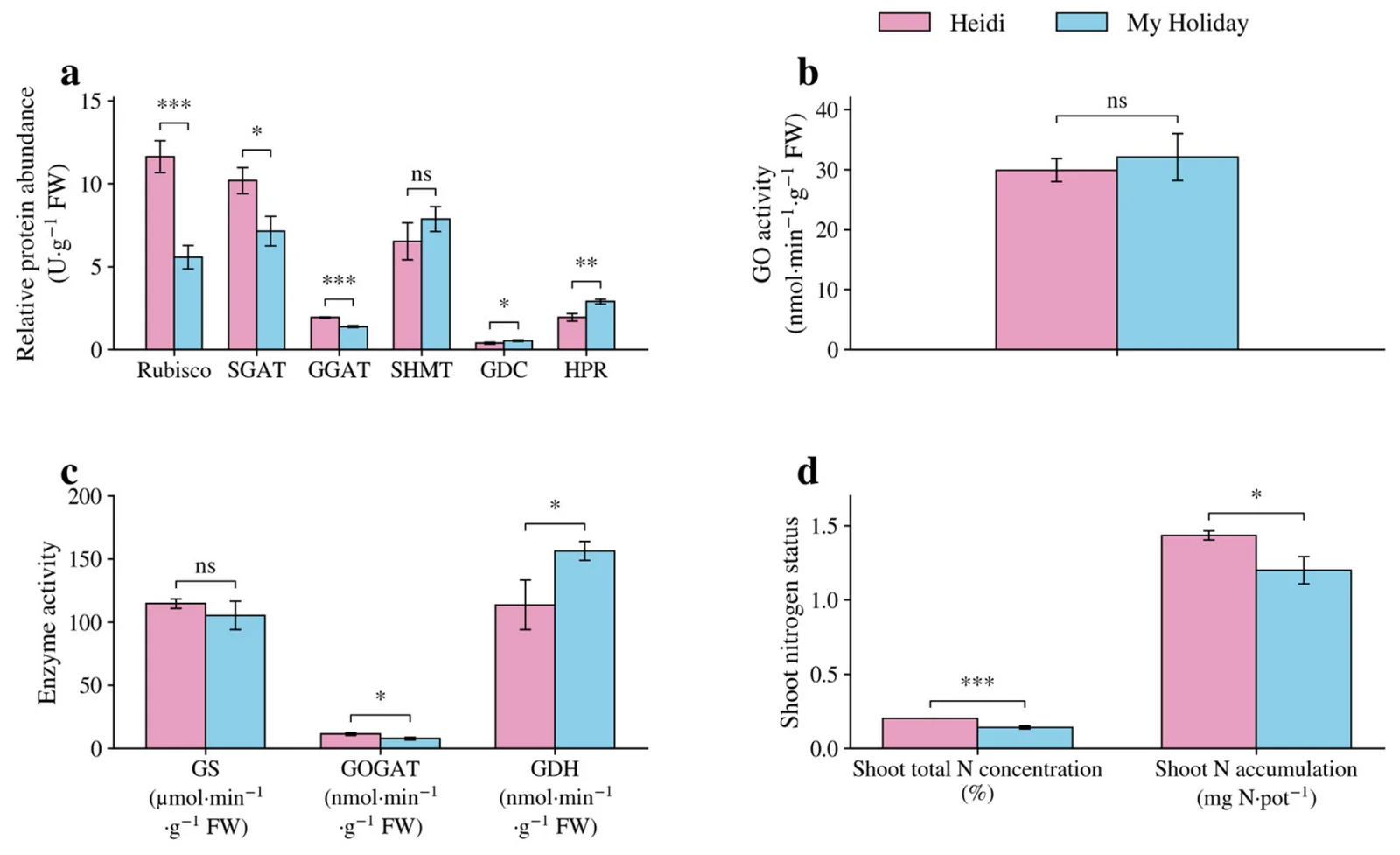
Photorespiration- and nitrogen-metabolism-related measurements in ‘Heidi’ and ‘My Holiday’ leaves under baseline growth conditions. (**a**) ELISA-derived relative protein abundance of Rubisco, SGAT, GGAT, SHMT, GDC, and HPR; (**b**) GO activity; (**c**) GS, GOGAT, and GDH activities; and (**d**) shoot total N concentration and shoot N accumulation. Values are means ± SD of three biological replicates. ns, *, **, and *** indicate *p* ≥ 0.05, *p* < 0.05, *p* < 0.01, and *p* < 0.001, respectively (two-sided independent-samples *t*-test).

The biochemical measurements did not show a uniformly coordinated cultivar response (Figure 2a–c). Among the ELISA-derived measurements, ‘Heidi’ had higher relative protein abundance of Rubisco, SGAT, and GGAT than ‘My Holiday’, whereas the relative protein abundances of GDC and HPR were higher in ‘My Holiday’. SHMT relative protein abundance did not differ significantly between the cultivars, and GO activity also showed no significant cultivar difference. Among the nitrogen-metabolism enzymes, GOGAT activity was higher in ‘Heidi’, GDH activity was higher in ‘My Holiday’, and GS activity did not differ significantly. ‘Heidi’ nevertheless had higher shoot total N concentration and shoot N accumulation than ‘My Holiday’ (Figure 2d). Overall, the measurements showed component-specific cultivar differences across photorespiration- and nitrogen-metabolism-related traits.

### 3.4. Effects of Nitrogen Form and Application Rate on Photorespiration and PFP_N_

**Figure 3:**
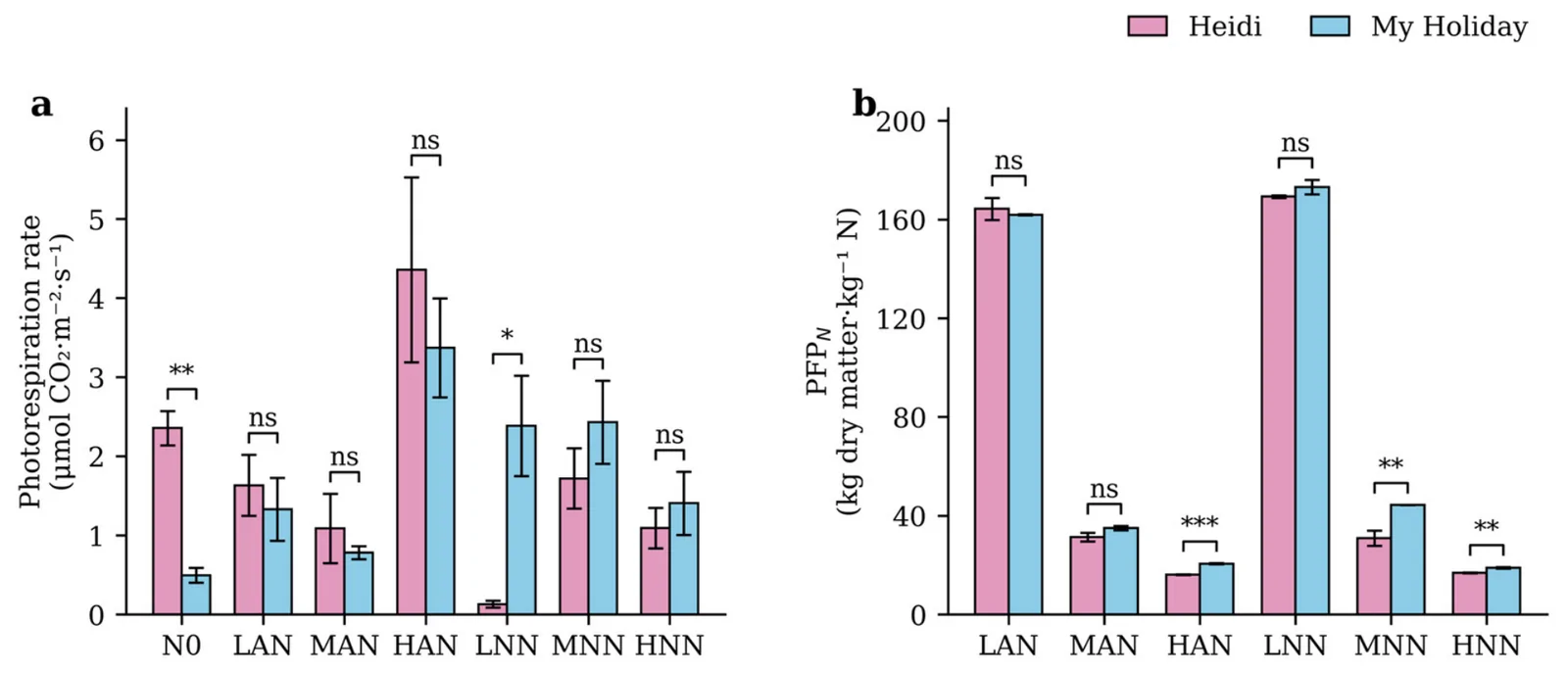
Effects of nitrogen form and application rate on photorespiration rate and partial factor productivity of applied nitrogen (PFP_N_) in ‘Heidi’ and ‘My Holiday’. (**a**) Photorespiration rate under the seven nitrogen treatments; (**b**) PFP_N_ under the six fertilized treatments. N0, zero-N control; LAN, MAN, and HAN, ammonium N at 1, 5, and 10 g N·m^−2^, respectively; LNN, MNN, and HNN, nitrate N at 1, 5, and 10 g N·m^−2^, respectively. Values are means ± SD of three independent pots. Within each treatment, ns, *, **, and *** indicate Holm-adjusted *p* ≥ 0.05, *p* < 0.05, *p* < 0.01, and *p* < 0.001, respectively, based on two-sided independent-samples *t*-tests. PFP_N_ was not calculated for N0 because no N was applied.

Nitrogen treatment altered the relative photorespiratory responses of the two cultivars (Figure 3a; Table S1). Across all seven treatments, the main effect of nitrogen treatment was significant (*F*_6,28_ = 24.56, *p* < 0.001), whereas the main effect of cultivar was not significant (*F*_1,28_ = 0.03, *p* = 0.867). The cultivar × nitrogen-treatment interaction was significant (*F*_6,28_ = 10.69, *p* < 0.001), indicating that the cultivar difference depended on the nitrogen treatment. Analysis of the six fertilized treatments showed that the cultivar × N form × N rate interaction was not significant for photorespiration rate (*F*_2,24_ = 1.82, *p* = 0.184), although several main effects and two-way interactions were significant (Table S1). Under N0, ‘Heidi’ had a higher photorespiration rate than ‘My Holiday’ (2.356 ± 0.218 vs. 0.493 ± 0.094 μmol CO_2_·m^−2^·s^−1^; Holm-adjusted *p* = 0.0012). In contrast, under LNN, the photorespiration rate was higher in ‘My Holiday’ than in ‘Heidi’ (2.383 ± 0.634 vs. 0.128 ± 0.043 μmol CO_2_·m^−2^·s^−1^; Holm-adjusted *p* = 0.021). No significant cultivar difference was detected under the other fertilized treatments after Holm adjustment.

PFP_N_ decreased with increasing N rate in both cultivars (Figure 3b). The three-way analysis showed a significant cultivar × N form × N rate interaction for PFP_N_ (*F*_2,24_ = 8.44, *p* = 0.0017; Table S1), indicating that the cultivar difference depended jointly on nitrogen form and application rate. ‘My Holiday’ had higher PFP_N_ than ‘Heidi’ under HAN, MNN, and HNN, whereas the cultivar differences under LAN, MAN, and LNN were not significant after Holm adjustment. Under LNN, PFP_N_ was 169.28 ± 0.50 kg shoot dry matter·kg^−1^ N in ‘Heidi’ and 173.10 ± 2.91 kg shoot dry matter·kg^−1^ N in ‘My Holiday’ (Holm-adjusted *p* = 0.177). Thus, the higher photorespiration rate of ‘My Holiday’ under LNN was not accompanied by a significant cultivar difference in PFP_N_.

### 3.5. Identification of PpSHMT and Biochemical Responses Following Transient Delivery in Tobacco

Transcriptome analysis identified a differentially expressed transcript annotated as serine hydroxymethyltransferase 4 (*SHM4*) in the photorespiration pathway (Table 4). The transcript showed a log_2_ fold change of 3.37 in ‘Heidi’ relative to ‘My Holiday’, and qRT-PCR independently confirmed higher *PpSHMT* transcript abundance in ‘Heidi’, with an approximately 3.3-fold difference relative to ‘My Holiday’ (*p* < 0.01; Figure S1a). However, this transcriptional difference was not accompanied by a significant difference in SHMT relative protein abundance between the two cultivars (Figure 2a). Thus, the higher transcript abundance of *PpSHMT* in ‘Heidi’ did not correspond to a detectable increase in bulk-leaf SHMT relative protein abundance. The *PpSHMT* coding sequence was amplified and sequenced, providing the molecular material required for the subsequent transient-delivery experiment in tobacco (Figure S1b,c).

The full-length *PpSHMT* coding sequence was inserted downstream of the CaMV 35S promoter in pBI121 (Figure S2a). Diagnostic restriction digestion produced the expected band pattern, supporting the presence of the *PpSHMT* insert in the recombinant construct (Figure S2b). PCR amplification from infiltrated tobacco leaves detected the expected *PpSHMT* fragment in TG samples (Figure S2c), supporting delivery of pBI121-PpSHMT into the leaf tissue. Together, these analyses supported construct assembly and delivery but did not quantify *PpSHMT* transcript or protein abundance.

**Figure 4:**
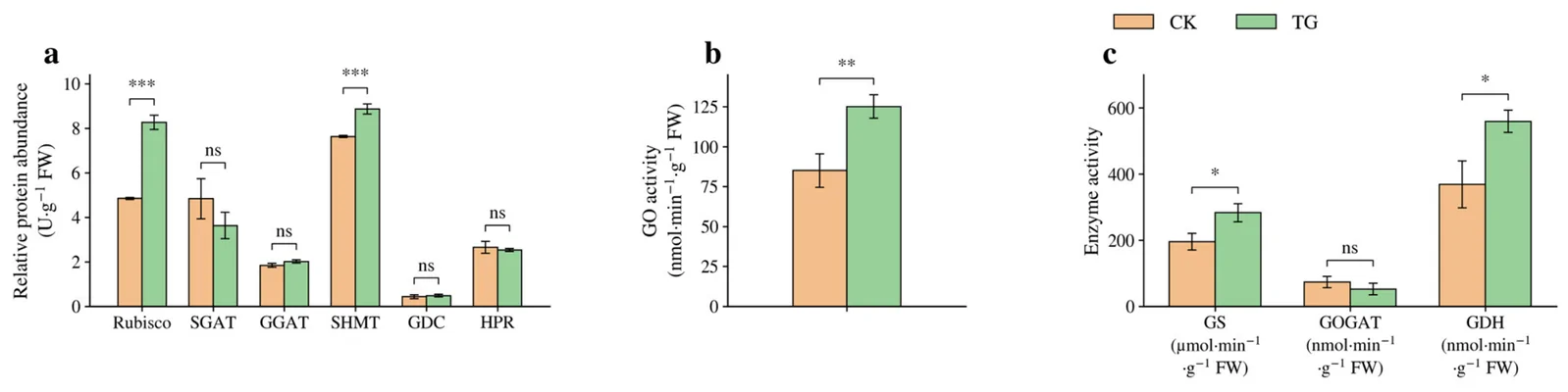
Photorespiration- and nitrogen-metabolism-related measurements in tobacco leaves. CK, untreated wild-type *N. tabacum* L. cv. K326 leaves; TG, leaves infiltrated with *A. tumefaciens* GV3101 carrying pBI121-PpSHMT. (**a**) ELISA-derived relative protein abundance of Rubisco, SGAT, GGAT, SHMT, GDC, and HPR; (**b**) GO activity; and (**c**) GS, GOGAT, and GDH activities. Values are means ± SD of three biological replicates. ns, *, **, and *** indicate *p* ≥ 0.05, *p* < 0.05, *p* < 0.01, and *p* < 0.001, respectively, based on two-sided independent-samples *t*-tests. No empty-vector infiltration control was included in this experiment.

Leaves infiltrated with *A. tumefaciens* GV3101 carrying pBI121-PpSHMT showed significantly higher ELISA-derived relative protein abundance of Rubisco and SHMT than untreated wild-type leaves (Figure 4a). GO, GS, and GDH activities were also significantly higher in the infiltrated leaves (Figure 4b,c), whereas no significant differences were detected in the relative protein abundance of SGAT, GGAT, GDC, or HPR or in GOGAT activity. These differences were observed after transient delivery of pBI121-PpSHMT; however, their specificity to *PpSHMT* could not be determined without an empty-vector control.

## 4. Discussion

In this study, ‘Heidi’ and ‘My Holiday’ were selected as contrasting Kentucky bluegrass (*P. pratensis* L.) cultivars based primarily on their respective highest and lowest photorespiration intensities, while their absolute photorespiration rates also differed significantly. The use of differences in net photosynthetic rates under 2% and 21% O_2_ to estimate photorespiration is well established for C_3_ species [22,32] and provided a consistent basis for cultivar screening. Under baseline conditions, ‘Heidi’ had higher NDVI, shoot N concentration, and shoot N accumulation than ‘My Holiday’, consistent with the reported association between NDVI and plant N status [19,20]. The cultivars also differed in upper-epidermal cell density, epidermal thickness, and abaxial stomatal density [33]. Although such anatomical traits can influence gas exchange under some environmental conditions [34,35,36], pigment contents and instantaneous gas-exchange variables did not differ significantly between the cultivars. Because the measured gas-exchange variables were unchanged, the functional significance of these anatomical differences remains uncertain. Similarly, the biochemical measurements showed component-specific and nonuniform responses: Rubisco relative protein abundance and GOGAT activity were higher in ‘Heidi’, whereas other photorespiration- and nitrogen-metabolism-related measurements showed either opposite patterns or no significant difference. These results indicate that contrasting photorespiration intensities were associated with selected structural, biochemical, and shoot-N traits but did not produce uniform activation of the photorespiration–nitrogen-metabolism network [14,37,38,39].

The nitrogen-treatment experiment further showed that the relationship between photorespiration and nitrogen productivity was conditional on nitrogen form and application rate. Under low nitrate supply, ‘My Holiday’ had a significantly higher photorespiration rate than ‘Heidi’, whereas PFP_N_ did not differ significantly between the cultivars. More broadly, the significant treatment interactions indicated that cultivar differences were not maintained consistently across nitrogen conditions. Nitrate assimilation requires substantial reducing power because nitrate and nitrite reduction use cellular reducing equivalents, whereas photorespiration affects redox balance and releases NH_3_ during glycine decarboxylation. The released NH_3_ is subsequently reassimilated through the GS/GOGAT cycle, providing a plausible metabolic connection among nitrate supply, photorespiration, and nitrogen assimilation [2,13]. Future measurements of nitrate-reductase activity, cellular NAD(H)/NADP(H) status, and carbon–nitrogen metabolic fluxes could clarify the direction and metabolic basis of this interaction. In addition, PFP_N_ was calculated as shoot dry matter produced per unit of applied elemental N because perennial turfgrass is managed primarily for vegetative biomass and turf performance rather than grain yield [4]. It should therefore be interpreted as an agronomic indicator of vegetative biomass productivity, distinct from physiological nitrogen-use efficiency, fertilizer-N recovery efficiency, and the grain-yield-based indices commonly applied to cereal crops [29].

Comparative transcriptomic analysis identified *SHM4*, hereafter referred to as *PpSHMT*, as a candidate transcript associated with the contrasting cultivar phenotypes. *PpSHMT* transcript abundance was substantially higher in ‘Heidi’ than in ‘My Holiday’ (log_2_FC = 3.37), and qRT-PCR confirmed the direction of this expression difference. However, bulk-leaf SHMT relative protein abundance did not differ significantly between the cultivars. Thus, the transcriptional difference was not accompanied by a corresponding difference in the ELISA-derived SHMT measurement. SHMT functions at the interface of photorespiration and serine–glycine–one-carbon metabolism, and plant SHMT families contain multiple isoforms with distinct subcellular localizations and physiological roles [40,41,42,43]. The transcript detected in the present analysis represents one SHMT family member, whereas the bulk-leaf ELISA measurement may include contributions from several isoforms. The observed discrepancy may also reflect post-transcriptional regulation, protein turnover, post-translational modification, subcellular targeting, or feedback associated with cellular glycine and serine pools. Isoform-specific expression analysis, targeted protein quantification, subcellular localization, and amino acid profiling will therefore be necessary to determine how *PpSHMT* transcription is related to SHMT protein accumulation and photorespiratory metabolism in Kentucky bluegrass.

**Figure 5:**
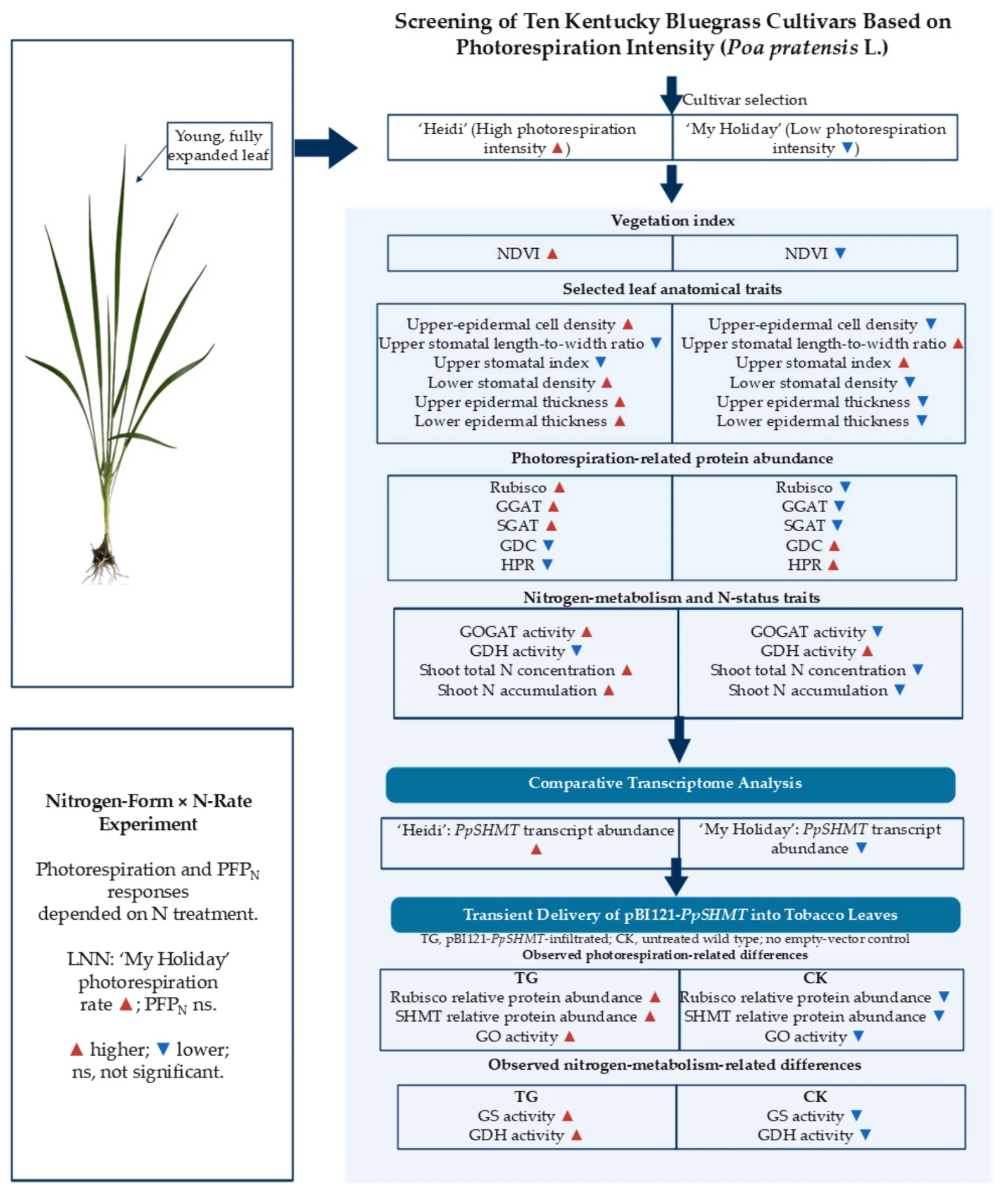
Summary of the experimental workflow and the principal associations among photorespiration intensity, selected anatomical and biochemical traits, nitrogen-treatment responses, PFP_N_, and *PpSHMT*. ‘Heidi’ and ‘My Holiday’ were selected based on their contrasting photorespiration intensities. In the tobacco experiment, TG denotes leaves infiltrated with *A. tumefaciens* GV3101 carrying pBI121-PpSHMT, whereas CK denotes untreated wild-type *N. tabacum* L. cv. K326 leaves.

PCR detected the expected *PpSHMT* fragment in infiltrated leaves, but transcript and protein expression were not quantified. Because CK consisted of untreated leaves and no empty-vector control was included, infiltration- and vector-related responses could not be separated from *PpSHMT*-specific effects. The biochemical changes should therefore be considered preliminary. Future experiments should include untreated, empty-vector, and pBI121-PpSHMT treatments, together with direct expression verification. Stable transformation and loss-of-function approaches, including RNA interference or CRISPR/Cas-based gene editing, will also be required to determine whether *PpSHMT* directly regulates photorespiration–nitrogen metabolism interactions. The experimental workflow and principal associations are summarized in Figure 5.

## 5. Conclusions

Kentucky bluegrass cultivars ‘Heidi’ and ‘My Holiday’ differed in photorespiration intensity, selected physiological, anatomical, and biochemical traits, and shoot N status. However, the nitrogen-treatment experiment showed that the relationship between photorespiration and nitrogen productivity depended on nitrogen form and application rate. Under low nitrate supply, the higher photorespiration rate of ‘My Holiday’ was not accompanied by a significant cultivar difference in PFP_N_. These results support a conditional association between photorespiration and nitrogen metabolism.

Comparative transcriptomic analysis and qRT-PCR identified *PpSHMT* as a candidate transcript associated with the contrasting cultivar phenotypes, although its higher transcript abundance in ‘Heidi’ was not accompanied by greater bulk-leaf SHMT relative protein abundance. Transient delivery of pBI121-PpSHMT into tobacco leaves was associated with changes in selected biochemical measurements, but the absence of an empty-vector control and direct verification of *PpSHMT* expression prevents these responses from being attributed specifically to *PpSHMT*. Future studies should combine appropriate infiltration controls, direct expression verification, and stable gain- and loss-of-function analyses to test the regulatory role of *PpSHMT*.

## Figures and Tables

**Table 1:** Details of Kentucky bluegrass cultivars used in this study [4].

Cultivar	Species	Source	Phenotypic Group
Blue ghost	*Poa pratensis X secunda*	DLF Pickseed, Halsey, OR, USA	Secunda hybrid
Comet	*P. pratensis*	Rutgers University, New Brunswick, NJ, USA	Compact
Geronimo	*P. pratensis*	DLF Pickseed, Halsey, OR, USA	Common
GreenStar	*P. pratensis*	DLF Pickseed, Halsey, OR, USA	Hybrid
Fielder	*P. pratensis*	DLF Pickseed, Halsey, OR, USA	Shamrock
Heidi	*P. pratensis*	DLF Pickseed, Halsey, OR, USA	Compact
Jackpot	*P. pratensis*	Jacklin Simplot Seed Co., Post Falls, ID, USA	BVMG
Jackrabbit	*P. pratensis*	DLF Pickseed, Halsey, OR, USA	Julia
My Holiday	*P. pratensis*	Jacklin Simplot Seed Co., Post Falls, ID, USA	Hybrid
Park	*P. pratensis*	Rivards Turf&Forage, Grand Forks, ND, USA	Common (Midwest)

**Table 2:** Photorespiration rate and photorespiration intensity in ten Kentucky bluegrass cultivar leaves.

Cultivar	*Rp* (μmol CO_2_·m^−2^·s ^−1^)	Photorespiration Intensity (%)
Blue ghost	2.10 ± 0.13 ^bc^	14.68 ± 0.78 ^de^
Comet	1.41 ± 0.39 ^d^	12.29 ± 3.34 ^ef^
Geronimo	0.61 ± 0.09 ^e^	9.64 ± 1.41 ^f^
GreenStar	1.48 ± 0.31 ^d^	16.91 ± 3.01 ^d^
Fielder	2.27 ± 0.04 ^bc^	26.20 ± 0.39 ^bc^
Heidi	2.36 ± 0.22 ^b^	31.17 ± 2.75 ^a^
Jackpot	3.48 ± 0.20 ^a^	23.83 ± 1.29 ^c^
Jackrabbit	1.48 ± 0.06 ^d^	28.68 ± 0.88 ^ab^
My Holiday	0.49 ± 0.09 ^e^	6.05 ± 1.15 ^g^
Park	1.98 ± 0.10 ^c^	16.06 ± 0.81 ^d^

Notes: Data are presented as the mean ± standard deviation (SD). Within each column, means followed by different lowercase letters differ significantly according to Tukey’s HSD test following one-way analysis of variance (*p* < 0.05).

**Table 3:** Structural characteristics of the leaf epidermis and leaf anatomy in ‘Heidi’ and ‘My Holiday’.

Index	Epidermis Side	Cultivar		*t*	*p*
		‘Heidi’ (*n* = 3)	‘My Holiday’ (*n* = 3)		
Cell length-to-width ratio	Upper	14.00 ± 1.00	12.60 ± 1.58	1.298	0.264
	Lower	5.52 ± 1.01	7.83 ± 1.28	−2.449	0.070
Stomatal length-to-width ratio	Upper	3.69 ± 0.45	4.51 ± 0.20	−2.916	0.043
	Lower	3.11 ± 0.60	3.42 ± 0.49	−0.697	0.524
Stomatal index (%)	Upper	5.62 ± 1.15	15.38 ± 2.32	−6.532	0.003
	Lower	29.17 ± 6.31	27.15 ± 4.75	0.444	0.680
Cell density (units·mm^−2^)	Upper	161.67 ± 15.14	64.00 ± 5.29	10.545	0.004
	Lower	156.00 ± 37.04	106.33 ± 22.19	1.992	0.117
Stomatal density (units·mm^−2^)	Upper	9.67 ± 2.36	11.56 ± 1.17	−1.241	0.283
	Lower	62.33 ± 5.69	38.83 ± 3.25	6.213	0.003
Epidermal thickness (μm)	Upper	64.71 ± 14.71	33.05 ± 4.81	3.543	0.024
	Lower	59.96 ± 1.70	35.29 ± 10.40	4.054	0.015
Leaf thickness (μm)	/	401.82 ± 15.70	316.13 ± 83.94	1.738	0.157
Sclerenchyma thickness (μm)	/	85.45 ± 2.59	64.70 ± 20.11	1.772	0.151
Vascular bundle area (μm^2^)	/	21,865.05 ± 8132.62	12,791.05 ± 2497.07	1.847	0.138

Note: Data are presented as the mean ± standard deviation (SD) of three biological replicates. Fifty microscopic fields were examined for each cultivar as observational subsamples. Statistical comparisons were based on three biological replicates per cultivar (*n* = 3).

**Table 4:** Photorespiration-related transcripts differentially expressed between ‘Heidi’ and ‘My Holiday’.

Gene ID	Annotation	Gene Symbol	‘Heidi’ Leaf TPM	‘My Holiday’ Leaf TPM	log_2_FC	Regulation	*p* Value	FDR	Significant
TRINITY_DN13353_c1_g1	serine hydroxymethyltransferase 4 [*Brachypodium distachyon*]	SHM4	15.56	1.69	3.37	Upregulated	5.05 × 10^−23^	1.64 × 10^−21^	Yes

Note: TPM, transcripts per million; log_2_FC, log_2_ fold change; FDR, false discovery rate.

## Data Availability

Data can be obtained from the corresponding author upon reasonable request.

## Supplementary Materials

The following supporting information can be downloaded at: https://www.mdpi.com/article/10.3390/biology15181628/s1, Figure S1: qRT-PCR analysis and molecular characterization of *PpSHMT* in Kentucky bluegrass cultivars ‘Heidi’ and ‘My Holiday’. Figure S2: Construction and PCR detection of pBI121-*PpSHMT* in infiltrated tobacco leaf tissue. Table S1: Analysis of variance for photorespiration rate and partial factor productivity of applied nitrogen (PFP_N_).
