# Supplementary material for: Photorespiration Is Associated with Nitrogen Metabolism and Nitrogen Productivity in Kentucky Bluegrass
Source: Biology (Basel). 2026 Sep 16;15(18):1628. doi: 10.3390/biology15181628 (PMC13603846; doi:10.3390/biology15181628)
Supplement: Supplementary file 1 [file biology-15-01628-s001.zip › biology-4496695-supplementary.pdf]

Table S1. Analysis of variance for photorespiration rate and partial factor productivity of applied nitrogen (PFP_N).

Dataset and response variable	Effect	F	p
Seven treatments: photorespiration rate	Cultivar	$F_{1,28} = 0.03$	0.867
	N treatment	$F_{6,28} = 24.56$	<0.001
	Cultivar × N treatment	$F_{6,28} = 10.69$	<0.001
Six fertilized treatments: photorespiration rate	Cultivar	$F_{1,24} = 2.56$	0.123
	N form	$F_{1,24} = 10.46$	0.0035
	N rate	$F_{2,24} = 18.35$	<0.001
	Cultivar × N form	$F_{1,24} = 21.50$	<0.001
	Cultivar × N rate	$F_{2,24} = 4.72$	0.0186
	N form × N rate	$F_{2,24} = 39.26$	<0.001
	Cultivar × N form × N rate	$F_{2,24} = 1.82$	0.184
Six fertilized treatments: PFP_N	Cultivar	$F_{1,24} = 44.92$	<0.001
	N form	$F_{1,24} = 41.91$	<0.001
	N rate	$F_{2,24} = 22695.08$	<0.001
	Cultivar × N form	$F_{1,24} = 13.37$	0.0012
	Cultivar × N rate	$F_{2,24} = 13.78$	<0.001
	N form × N rate	$F_{2,24} = 15.63$	<0.001
	Cultivar × N form × N rate	$F_{2,24} = 8.44$	0.0017

Note: N0 was included only in the two-way analysis of photorespiration rate. The three-way analyses included the six fertilized treatments.

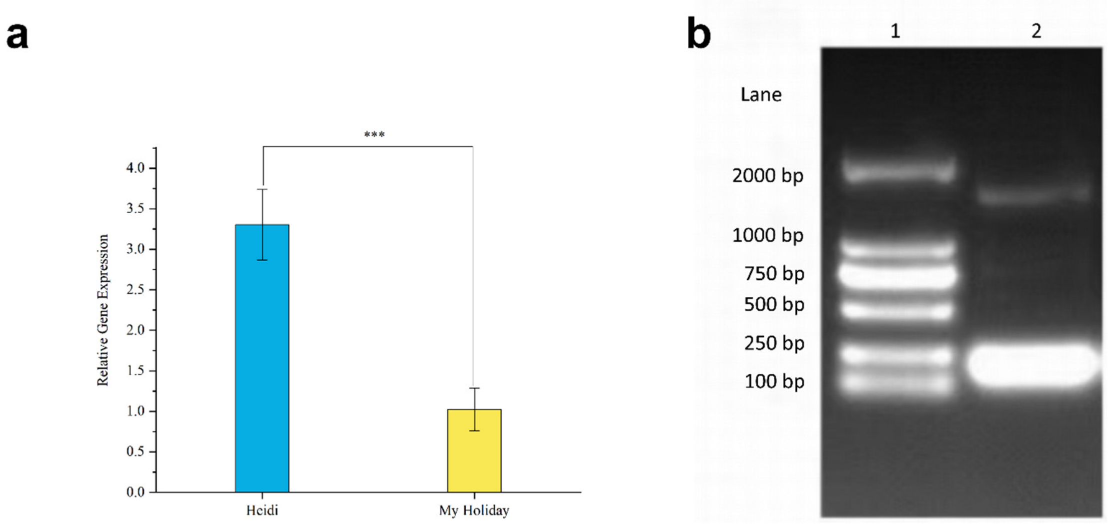

c

```
1     GGATCCATGGCCGCGCAGCCACCCATGGTGCGCGCCGCTTTACACCCACACCCCATCACCGCTCCTCTCCCCAGTCACCATCACCCCTCCCCTCTCCGGCTCCCGCTCCTCCGCGCCGCC
1      G  S  M  A  A  Q  P  P  M  V  R  A  A  L  H  P  H  P  I  T  A  P  L  P  S  H  H  H  P  S  P  L  R  L  P  L  L  R  A  A
121   GCCCGCCCCCTCCGCCTCCACGCCATCTCCACCGACTCCACCCCAGCCCCCAACGCCGCCCCCATGGACGCCGCCGCCCCGATAGACGCTGTGGCATCCTGGGGCCTAACCCCACTATCG
41     A  R  P  L  R  L  H  A  I  S  T  D  S  T  P  A  P  N  A  A  P  M  D  A  A  A  P  I  D  A  V  A  S  W  G  L  T  P  L  S
241   GAGGCCGACCCGGAGGTGTACGACCTAATCGAGCGCGAGAAGCGCCGCCAGCACACGGGCATCGAGCTAATCGCCTCGGAGAACTTCACCTCCCTCGCCGTGATGGAGGCCCTGGGCTCC
81     E  A  D  P  E  V  Y  D  L  I  E  R  E  K  R  R  Q  H  T  G  I  E  L  I  A  S  E  N  F  T  S  L  A  V  M  E  A  L  G  S
361   CCGCTCACCAACAAGTACTCGGAGGGCATGCCGGGCGCGCGCTACTACGGCGGGAACGAGGTCATCGACCAGGTCGAAAACCTCTGCCGCGCCCGGGCCCTCCAGGCCTACCGCCTCGAC
121    P  L  T  N  K  Y  S  E  G  M  P  G  A  R  Y  Y  G  G  N  E  V  I  D  Q  V  E  N  L  C  R  A  R  A  L  Q  A  Y  R  L  D
481   GCGGCGCGCTGGGGCGTCAACGTGCAGCCCTACTCCGGCTCCCCGGCCAACTTCGCCGCGTACACCGGCCTGCTCCAGCCCCACGACCGCATCATGGGGCTCGACCTGCCGTCCGGCGGG
161    A  A  R  W  G  V  N  V  Q  P  Y  S  G  S  P  A  N  F  A  A  Y  T  G  L  L  Q  P  H  D  R  I  M  G  L  D  L  P  S  G  G
601   CACCTCACCCATGGGTATTACACGGCCGGGGGGAAGAAGATTTCGGCAACCTCGATATACTTCGAGAGCTTGCCGTATAAGGTGAGCTCTGAGACTGGGTATGTGGATTACGATAAGCTT
201    H  L  T  H  G  Y  Y  T  A  G  G  K  K  I  S  A  T  S  I  Y  F  E  S  L  P  Y  K  V  S  S  E  T  G  Y  V  D  Y  D  K  L
721   GAGGAGAAGGCCATGGATTTCCGCCCCAAGCTTATTATCTGCGGCGGGAGCGCCTACCCGCGGGACTGGGATTACGCCAGGTTCAGGGCCATCGCCGACAAGTGTGGCGCCTTGTTGCTC
241    E  E  K  A  M  D  F  R  P  K  L  I  I  C  G  G  S  A  Y  P  R  D  W  D  Y  A  R  F  R  A  I  A  D  K  C  G  A  L  L  L
841   TGTGACATGGCTCATATCAGCGGCCTCGTTGCCGCGCAGGAGGCTGCGAATCCTTTTGAGTATTGTGATGTGGTGACCACCACCACCCACAAGAGTCTCCGGGGGCCAAGGTCTGGTATG
281    C  D  M  A  H  I  S  G  L  V  A  A  Q  E  A  A  N  P  F  E  Y  C  D  V  V  T  T  T  T  H  K  S  L  R  G  P  R  S  G  M
961   ATATTCTACAGGAAAGGCCCGAAGCCTCCCAAAAAGGGACAGCCCGACAATGCTCTTTACGACTATGAGGACAAGATCAACTTTGCCGTGTTTCCATCACTTCAGGGCGGCCCTCACAAC
321    I  F  Y  R  K  G  P  K  P  P  K  K  G  Q  P  D  N  A  L  Y  D  Y  E  D  K  I  N  F  A  V  F  P  S  L  Q  G  G  P  H  N
1081  CACCAGATTGGTGCCCTGGCAGTTGGCCTGAAGCAGGCCATGTCACCTGGATTCAGGGCCTATATTCAGCAGGTCAAGGCCAATGCTGTTGCCGTTGCAGACCATCTGATGAGTAAGGGC
361    H  Q  I  G  A  L  A  V  G  L  K  Q  A  M  S  P  G  F  R  A  Y  I  Q  Q  V  K  A  N  A  V  A  V  A  D  H  L  M  S  K  G
1201  TACAAGTTGGTTACTGATGGAACAGAAAACCACCTTGTTCTCTGGGACCTTCGCCCTCTTGGCTTGTCCGGTAACAAAGTTGAGAAAGTATGTGATCTGAGCAGCATTACACTCAACAAA
401    Y  K  L  V  T  D  G  T  E  N  H  L  V  L  W  D  L  R  P  L  G  L  S  G  N  K  V  E  K  V  C  D  L  S  S  I  T  L  N  K
1321  AATGCCGTCTTTGGTGACAGCAGTGCATTGGCGCCTGGTGGTGTCCGAATTGGTACACCAGCGATGACCTCGAGGGGCTTGGGTTGAGGTACCGAGCTC
441    N  A  V  F  G  D  S  S  A  L  A  P  G  G  V  R  I  G  T  P  A  M  T  S  R  G  L  G  *  G  T  E  L
```

Supplementary Figure S1. qRT-PCR analysis and molecular characterization of *PpSHMT* in Kentucky bluegrass cultivars ‘Heidi’ and ‘My Holiday’. (a) Relative PpSHMT transcript abundance determined by qRT-PCR using *Actin* as the reference gene. Values are presented as the mean ± SD of three biological replicates, with three technical replicates per biological sample. Statistical significance was assessed using a two-sided independent-samples *t-test* based on ΔCt values (** $p < 0.01$). (b) PCR amplification of the *PpSHMT* coding sequence. Lane 1, AL2000 DNA marker; lane 2, amplified *PpSHMT* product. (c) Nucleotide and deduced amino acid sequences of *PpSHMT*. The coding sequence begins with the start codon ATG and terminates with the stop codon TGA.

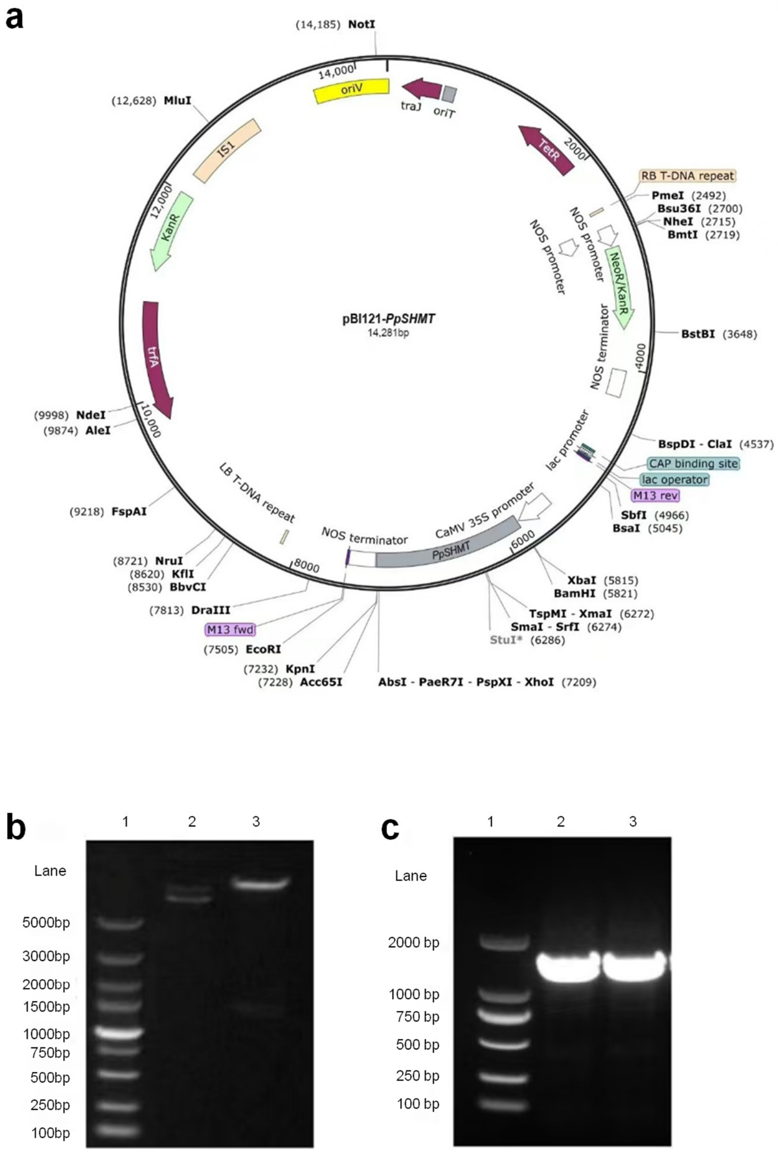

Supplementary Figure S2. Construction and PCR detection of pBI121-*PpSHMT* in infiltrated tobacco leaf tissue. (a) Map of the pBI121-*PpSHMT* recombinant construct. (b) Diagnostic restriction digestion of pBI121-*PpSHMT* using BamHI and KpnI. Lane 1, AL5000 DNA marker; lane 2, undigested recombinant plasmid; lane 3, restriction-digested recombinant plasmid. (c) PCR detection of the *PpSHMT* fragment in infiltrated tobacco leaf tissue. Lane 1, AL2000 DNA marker; lanes 2 and 3, PCR products amplified from two tobacco leaf samples infiltrated with Agrobacterium tumefaciens GV3101 carrying pBI121-*PpSHMT*. No untreated CK or no-template negative-control lane is shown in panel (c).
